# Molecular Characterisation of Faecal Bacterial Assemblages Among Four Species of Syntopic Odonates

**DOI:** 10.1007/s00248-023-02328-1

**Published:** 2023-12-18

**Authors:** A. Morrill, M. R. Forbes, E. J. Vesterinen, M. Tamminen, I. E. Sääksjärvi, K. M. Kaunisto

**Affiliations:** 1 Department of Biology, Carleton University, Ottawa, Ontario Canada; 2 Department of Biology, University of Turku, Turku, Finland; 3 Biodiversity Unit, University of Turku, Turku, Finland

**Keywords:** Metabarcoding, Faecal DNA, Damselfly, Bacterial assemblages, Biodiversity, Microbiome

## Abstract

Factors such as host species, phylogeny, diet, and both timing and location of sampling are thought to influence the composition of gut-associated bacteria in insects. In this study, we compared the faecal-associated bacterial taxa for three *Coenagrion* and one *Enallagma* damselfly species. We expected high overlap in representation of bacterial taxa due to the shared ecology and diet of these species. Using metabarcoding based on the 16S rRNA gene, we identified 1513 sequence variants, representing distinct bacterial ‘taxa’. Intriguingly, the damselfly species showed somewhat different magnitudes of richness of ZOTUs, ranging from 480 to 914 ZOTUs. In total, 921 (or 60.8% of the 1513) distinct ZOTUs were non-shared, each found only in one species, and then most often in only a single individual. There was a surfeit of these non-shared incidental ZOTUs in the *Enallagma* species accounting for it showing the highest bacterial richness and accounting for a sample-wide pattern of more single-species ZOTUs than expected, based on comparisons to the null model. Future studies should address the extent to which faecal bacteria represent non-incidental gut bacteria and whether abundant and shared taxa are true gut symbionts.

**Graphical Abstract:**

Pictures of odonates adopted from Norske Art databank under Creative Commons License (CC BY 4.0).

**Figure:**
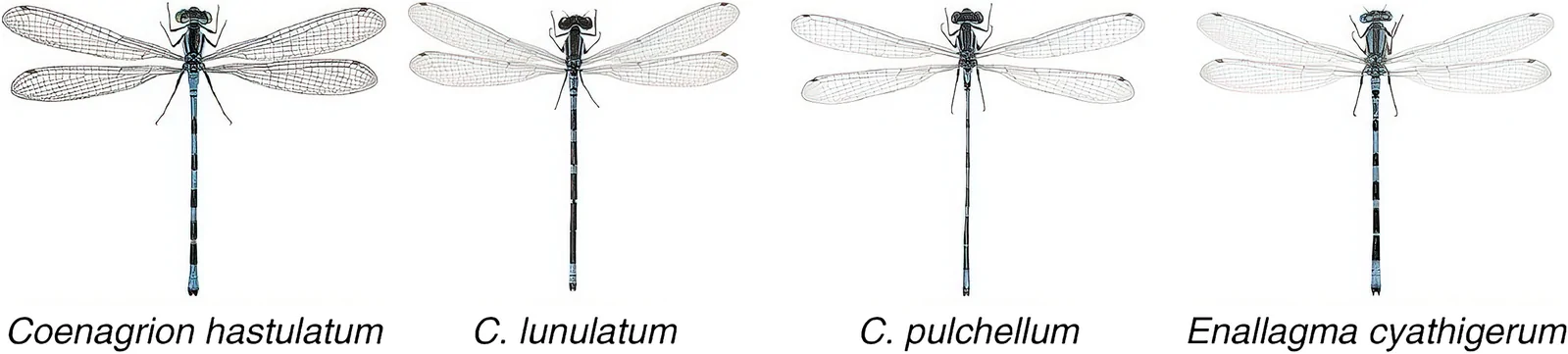

## Introduction

Insects are by far the most numerous animal species on Earth and play a significant role in our food security, economy, health care, and general well-being [1–4]. Despite the prominence of insects, we still know very little about the diversity, general composition, and functioning of insect gut-associated microbiota [5–7]. In recent years, however, there has been an increasing interest in studying insect-associated microbiomes [8]. Notably, we are beginning to comprehend the vital roles of insect microbiota in many important functions, such as enabling ligno- and cellulolytic digestion [9], protection against parasites [10], producing pheromones [11], and degrading and detoxicating secondary compounds of plants and pesticides [12, 13].

Many of the insect bacterial symbionts reside in the insect gut. Gut-associated bacteria have been implicated in providing nutritional benefits by provisioning essential amino acids [14] and by fixing nitrogen [15]. On another front, diet (e.g. herbivory versus predation) is thought to play a role in determining gut bacterial assemblages, such that herbivorous insects seem to have evolved to co-opt metabolic functions from symbiotic bacteria to deal with the digestion of tough plant tissues [16]. Researchers also expected gut-associated bacterial assemblages to be dependent on host taxonomy. However, insect taxonomy and phylogeny have explained little variation in bacterial assemblages [8].

Samples to assess microbiome assemblages can be taken from insect frass or faecal material, or even from insect products, such as honey [17]. Kaunisto, Roslin, Sääksjärvi, and Vesterinen [18] showed that faecal pellets of dragonflies and damselflies could be examined with metabarcoding techniques and bioinformatic approaches to reveal taxa (even species) of invertebrate prey being consumed. Such information can be used for describing food web linkages and testing general hypotheses in behavioural and community ecology [e.g. 19]. In principle, faecal pellets also could be examined using similar genetic and bioinformatic approaches to identify bacteria. At least, these approaches are expected to be complementary to bacterial identification from gut dissections, and using metabarcoding, histological and culturing methods [19].

Recent developments in molecular methods and bioinformatics have made it possible for researchers to study gut-associated bacterial taxa and assemblages of insects. So far, microbiota has been investigated only for a few groups of insects, such as for herbivorous pest insects of commercial importance [20], as well as Odonates [21]. Previous research on Odonate gut bacterial assemblages suggests that a few bacteria are obligate gut symbionts; the bacterial assemblage is transient and dependent on locations and timing of insect sampling [21]. Life stage (larva vs. adult) is also an important determinant of bacterial taxa represented [22]. Furthermore, since Odonates are generalist predators [23], there can be a considerable degree of dietary overlap between sexes within a species and even between species [18, 24]. However, there can also be diet differentiation depending on where and when the dragonflies were sampled [23]. This dependence of diet on sample location or timing should have implications for the diversity of bacteria these predatory insects catch along with their prey insects.

Many of the bacterial species are expected to be incidental (present in only one or a few hosts of a single species or present in certain locales and not others). These incidentally sampled bacterial species likely have little functional or other ecological significance (though it is possible that some transient insect gut microbes could provide a benefit to their host [25, 26]). Other bacterial species are expected to be core species, found in many host individuals, and likely shared between host species because of shared ecology (e.g. diet) or shared functional significance (e.g. aiding in digestion). It is the explicit consideration of non-shared and shared bacterial species that bears on questions of diversity and differentiation of bacterial assemblages between species.

In this study, we compared faecal-associated bacterial taxa for three *Coenagrion* and one *Enallagma* species of damselflies. We expected that these damselfly species would show highly overlapping faecal bacterial assemblages, given similarities in their ecology and sampled life stages, and given that these are syntopic populations with identical availability of prey. We asked the following questions: first, are there differences in the number of distinct zero-radius operational taxonomic units (ZOTUs) between our focal species? Second, to what extent are any differences in this richness due to the inclusion of incidentally sampled non-shared ZOTUs present in only one or a few individuals of a given host species (apparently unique to those host species)? Third, are there any patterns in shared vs. non-shared ZOTUs in these four host species? Fourth, do the bacteria showing potential between-species variation in read abundances come from particular regions of the phylogeny, or are they dispersed throughout it? Finally, we address the extent to which different classes of bacteria (e.g. alpha-, beta-, and Gammaproteobacteria, Bacilli) are represented among the four species of damselflies and among non-shared and shared ZOTUs.

## Methods

To assess the faecal bacterial assemblages of damselflies, we targeted four predatory odonate species at a freshwater pond of approximately 600 m × 200 m (12 ha), located in Southern Finland (ETRS-TM35FIN N: 67118; E: 2460). On 1–2 June 2016, we collected 185 individuals (20–26 males and females from each species) for faecal DNA analysis. All our focal damselfly species belong to the family Coenagrionidae: *Coenagrion lunulatum* (Charpentier, 1840), *Coenagrion hastulatum* (Charpentier, 1825), *Coenagrion pulchellum* (Vander Linden, 1825), and *Enallagma cyathigerum* (Charpentier, 1840). Species identification of damselflies was based on current literature, e.g. [27]. These four target species were selected as they were the most common predatory species at the study site, based on pilot surveys (K. Kaunisto, pers. obs.). Only sexually mature individuals with adult colours and hardened wings were included in the study. According to a previous study [28], all four focal species feed mainly on dipteran prey by open foraging flights and by gleaning insects from vegetation.

Each damselfly was placed into a sterile 10-ml collection tube housing a piece of dampened paper towel to reduce desiccation risk. To allow for defecation, damselflies were kept in the tubes for the next 24 h (sufficient time for defecation to occur, according to [18]). After the live individuals had defecated into the tube, we froze the entire sample without removing the faeces or the damselfly. All faecal material was collected from the tubes with sterile forceps, after which the faeces were frozen in 15-ml Falcon tubes at −64 °C until further processing and analysis.

### Sample Processing and Molecular Analysis

Total DNA was extracted as described in a previous study using NucleoSpin Tissue XS Kit (product nr 740901, Macherey-Nagel, Düren, Germany) [28]. To characterize the bacterial assemblages of the focal species, we used established metabarcoding protocols for dragonflies building on earlier optimization [18, 28]. To amplify bacterial 16S rRNA gene (hypervariable region v4), we used primers 515F-Parada (also known as 515FB: 5′-GTG YCA GCM GCC GCG GTA A-3′; Parada et al. 2016) and 806R-Apprill (also known as 806RB: 5′-GGA CTA CNV GGG TWT CTA AT-3′; [29]). Each DNA sample was amplified in two separate reactions that were individually tagged and sequenced. The locus-specific PCR setup followed Kankaanpaa, Vesterinen, Hardwick, Schmidt, Andersson, Aspholm, Barrio, Beckers, Bety, Birkemoe, DeSiervo, Drotos, Ehrich, Gilg, Gilg, Hein, Hoye, Jakobsen, Jodouin, Jorna, Kozlov, Kresse, Leandri–Breton, Lecomte, Loonen, Marr, Monckton, Olsen, Otis, Pyle, Roos, Raundrup, Rozhkova, Sabard, Sokolov, Sokolova, Solecki, Urbanowicz, Villeneuve, Vyguzova, Zverev, and Roslin [30] and included 5 μl of 2× MyTaq HS Red Mix (Bioline, UK), 2.4 μl of H_2_O, 150 nM of each primer (two forward and two reverse primer versions; total primer mix concentration 600 nM), and 2 μl of DNA extract per each sample in 10 μl volume. Cycling conditions were 3 min at 95 °C, then 35 cycles of 45 s at 95 °C, 1 min at 50 °C, and 1 min 30 s at 72 °C, ending with 10 min at 72 °C. In the second PCR stage, the first PCR products were modified by attaching Illumina-specific adapters and sample-specific indices. For a reaction volume of 10 μl in the indexing PCR, we mixed 5 μl of MyTaq HS RedMix, 500 nM of each tagged and indexed primer (i7 and i5), and 3 μl of locus-specific PCR product from the first PCR phase. For this second PCR, we used the following protocol: initial denaturation for 3 min at 98 °C, then 15 cycles of 20 s at 95 °C, 15 s at 60 °C, and 30 s at 72 °C, followed by 3 min at 72 °C. All the indexed reactions were then pooled and purified using magnetic beads [31, 32].

Sequencing was done on an Illumina MiSeq v3 PE 2×300 (Illumina Inc., San Diego, CA, USA) run, including the PhiX control library by the Turku Centre for Biotechnology, Turku, Finland. After sequencing, the reads were demultiplexed into each original sample and uploaded onto CSC servers (IT Center for Science, https://www.csc.fi/) for bioinformatic analysis. Paired-end reads (13,027,754) were merged and trimmed for quality using 64-bit *vsearch* version 2.14.2 [33] command ‘fastq_mergepairs’ with the default options and ‘fastq_allowmergestagger’. Primers were removed from the merged reads (11,179,018) using software *cutadapt* version 1.14 (Martin 2011) with 20% mismatch rate, minimum length of 240 bp and truncate length of 270 bp (the excess nucleotides were trimmed from 3′ end). Trimmed reads (11,050,385) were then collapsed into unique sequences (singletons removed) with command ‘fastx_uniques’ and option ‘minuniquesize’ set to 10 (49,832 uniques retrieved). Finally, reads were corrected for point errors to obtain an accurate set of amplicon sequences (=denoised) and filtered of chimeric amplicons (=chimeras were removed) resulting in 3803 ZOTUs (‘ZOTU’, ‘zero-radius OTU’) through command ‘unoise3’ using USEARCH version 11.0.667 with settings *minsize* = 8 and *unoise_alpha* = 2. The median and mean length of ZOTUs was 253 bp (SD ± 2.50 bp). Then ZOTUs were mapped back to the original trimmed reads with command ‘usearch_global’ to establish the total number of reads in each sample using *vsearch*. We were able to map 10,627,197 of 11,050,385 (96.17%) to our original samples. The ZOTUs (sequence variants) were assigned to taxa using 16 RDP database with SINTAX (Edgar, 2010) probabilistic algorithm implemented in *vsearch*. The database ‘16S RDP training set v18’ (21k seqs) was downloaded from the usearch website (https://drive5.com/usearch/manual/sintax_downloads.html; accessed 19th April 2023). For the chosen database, the genus level is the lowest taxonomic level. For any taxonomic level, we only accepted assignations with 100% probability. The data was further filtered to remove artefacts, spurious reads, and non-targets based on information on the numerous control samples, technical replicates, and taxonomy. First, we removed those ZOTUs from any sample that had fewer reads than extraction or PCR controls (9,833,618 reads retained). Then, we collapsed reads based on the taxonomy per each sample, that is, all the reads that were assigned to the same taxa per sample were summarized. Out of the 3803 ZOTUs, we identified 983 to genus, 1570 to family, 2002 to order, 3063 to class, 3319 to phylum, and 3482 to domain level. From the total ~10M reads, we identified 4.0M to genus, 4.4M to family, 8.5M to order, and 9.5M to the higher levels. Then, we removed taxa that were present in a sample by only one of the two replicates and finally summed the reads in both replicates (9,678,663 reads left). Then, to remove potentially leaked ‘tag-jumped’ reads from the data, we removed all taxa from the samples with less than 0.05% proportion of the total reads in one sample (9,636,233 reads saved). We removed all the taxa outside domains Bacteria or Archaea, as well as Class Chloroplast (9,006,117 reads passed the filtering). The non-targets included mainly plants (~6200 reads) and Fungi (~250 reads). Altogether 284,351 reads could not be assigned with the strict 100% probability threshold. Finally, very rare occurrences (sequence count < 20) were removed (9,004,996 final reads). Labelled raw FASTQ reads, ZOTUs, ZOTU assignations, and ZOTU tables are available in the Dryad Digital Repository: 10.5061/dryad.08kprr58q.

### Statistical Analyses

All analyses and data syntheses were performed using R version 4.1.2 [34], unless stated otherwise. Metabarcoding read abundance data was converted to ZOTU presence/absence data for the majority of analyses (i.e. for everything other than the core ZOTU relative read abundance heatmap and the DESeq2 analysis), to remain conservative. Analyses based on metabarcoding reads themselves generally assume variation in reads among samples could relate principally to meaningful biological differences among the samples. However, we found that the median percentage difference in read abundances for individual ZOTUs between paired samples was 50%; therefore, there is likely significant variation in read abundances not due to variation among individually sampled damselflies, contributing ‘noise’ to the abundance data. Some analyses were still conducted on read abundances themselves, as mentioned; this was done to aide both in demonstrating patterns and in interpretations. The results of these analyses should be considered with the above caveat.

The total number of different ZOTUs recorded in each damselfly species was counted (ZOTU richness). Accumulation curves were constructed to determine whether within-species observed ZOTU richness likely represented the diversity of ZOTUs present in the damselfly populations (i.e. whether the accumulation curves approached apparent horizontal asymptotes) by calculating the cumulative richness across samples over 200 randomizations of sample orderings. The extrapolated total (unobserved) ZOTU richness for each damselfly species was calculated using two different algorithms (Chao and first order jackknife; Colwell and Coddington [35]), implemented using the specpool() function from the R package vegan 2.6 [36]. We also calculated the proportion of observed ZOTUs within a species which were specific to that host species (for each damselfly species: [# of distinct species-specific ZOTUs]/[total # of ZOTUs observed for the species]), and the average number of damselfly individuals from which species-specific versus multi-species ZOTUs were sampled (for all ZOTUs observed in a damselfly species, calculated the number of individual damselflies in which the ZOTU was detected, then calculated averages for damselfly species-specific vs. multi-species ZOTUs).

Inter-individual ZOTU assemblage dissimilarities were quantified using Bray–Curtis dissimilarities based on presence-absence data ((A + B – 2 × J)/(A + B), where A and B are the number of ZOTUs present in each of the two focal samples, and J is the number of ZOTUs that are shared between them; [36]). Differences in ZOTU community composition between the four damselfly species were tested using PERMANOVA (10,000 permutations) and preceded by a test to ensure multivariate homogeneity of group variances (as PERMANOVA tests both the null hypotheses that group means and within-group variances are equal; betadisper() function in the vegan R package; [36]).

To test hypotheses relating to the relative frequencies of occurrences of ZOTUs in single or multiple damselfly species, null models were created by randomly permuting the occurrence matrix (presence/absences of ZOTUs across all samples) 1000 times, such that row and column sums are maintained (each ZOTU always present in the same number of damselfly specimens; each specimen always having the same number of distinct ZOTUs present). These constraints on matrix row and column sums were required to ensure that null model comparisons were meaningful, by maintaining both the relative rarities of the diverse ZOTUs as well as the observed relative ‘habitabilities’ of the damselfly faecal samples to microbes [37]. This was accomplished using the permatswap() function in the vegan package, implementing the quasi-swap permutation algorithm [36, 38]. These randomized matrices were used as null models to compare the expected and observed numbers of ZOTUs occurring in specimens from one, two, three, or four different damselfly species (for example, to ask: are faecal ZOTUs generally more or less species-specific than expected due to chance?), as well as the expected and observed number of ZOTUs specific to, or shared between, certain damselfly species (e.g. are the number of ZOTUs observed only in one damselfly species, or shared between two particular species, more or less than expected due to chance?). In other words, we explore evidence for a non-random assortment of faecal ZOTUs among damselfly species by considering frequencies of shared or species-specific ZOTUs across specimens. Ninety-five percent confidence intervals for values calculated from the null model randomizations (e.g. expected ZOTU richnesses) are the highest density continuous intervals. Visualization of the Venn diagram was aided in part through the use of the ggVennDiagram R package (version 1.2.0; Gao, Yu and Cai [39]).

While focusing on comparisons of non-shared versus shared faecal bacteria between the four species of damselfly, some analyses concerned specific ZOTUs (e.g. differences in focal ZOTU representation between species). The remaining tests focussed on the relative frequencies of species-specific versus multi-species ZOTUs in general.

To consider phylogeny explicitly, the sequences were aligned against the Silva SSURef_NR99_128_SILVA_07_09_16 database [40] using Sina version 1.7.2 [41]. A phylogenetic tree was constructed from the aligned ZOTU sequences using FastTree version 2.1.11 [42]. The ZOTU data was processed using phyloseq version 1.38 [43], and the phylogenetic tree was visualized using ggtree version 3.2.1 [44]. Differentially abundant ZOTUs were identified using DESeq2 using the damselfly species as the grouping variable [45]. This method applies generalized linear models with negative binomial responses to test for differences in the expected absolute read abundances between damselfly species for each ZOTU, incorporating data-driven priors and adjusting test *p*-values to account for multiple comparisons.

## Results

### Observed and Extrapolated ZOTU Richness for Each Damselfly Species

**Fig. 1:**
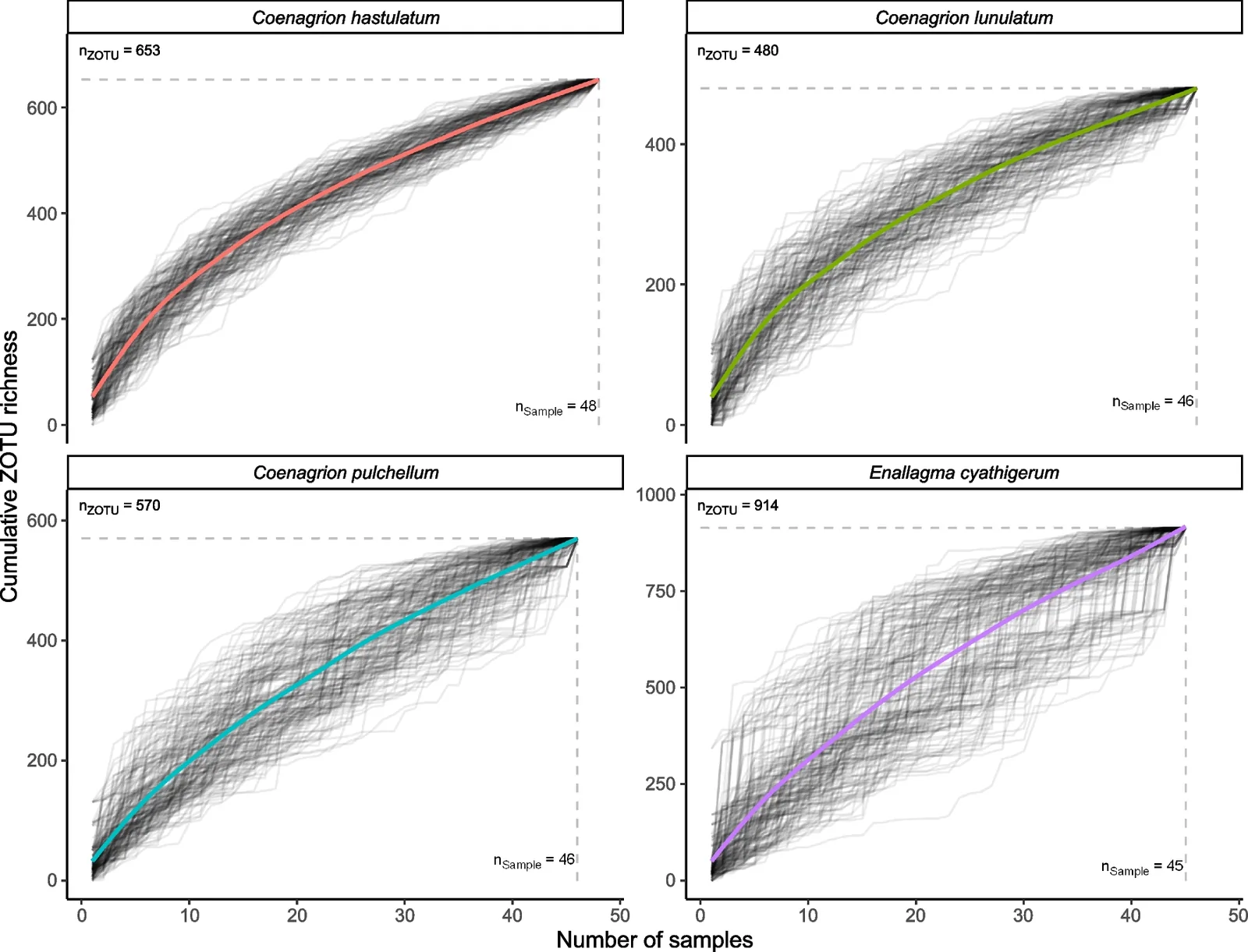
ZOTU accumulation curves for each damselfly species generated from 200 randomizations of the sample orderings. Individual accumulation curves from each random ordering are shown in translucent grey, while the smoothed average (thick, coloured) lines were fit using generalized additive models with a cubic regression spline on the sample size covariate (R function mcv∷gam()). Total observed ZOTU richnesses and sample sizes for each damselfly species are indicated with dashed lines and the annotated text. Note that the *Y*-axis ranges differ among the four panels

Accumulation curve saturation was not reached for any damselfly species, but the curves were in a decelerating phase for all species, suggesting that maximum numbers of distinct ZOTUs were not far off (Fig. 1). The pairs of extrapolated total ZOTU richness estimates (Chao and first order jackknife) are reported for each of the four species (Table 1). While there is insufficient information to confidently infer that total ZOTU richness differs among the four species, there is some evidence that *C. hastulatum* and particularly *E. cyathigerum* (the two species with the highest extrapolated richnesses) have faecal bacterial assemblages that are more ZOTU-rich than *C. lunulatum* (95% CIs derived from the standard errors are non-overlapping for the Chao, but not first order jackknife, estimates; Table 1). The number of unique ZOTUs (defined as being specific to a single damselfly species) recovered ranged from a low of 480 for 46 sampled *C. lunulatum* individuals to a high of 914 for 45 *E. cyathigerum* individuals (Table 1). The two remaining species, *C. hastulatum* and *C. pulchellum*, were intermediate in numbers of distinct ZOTUs recovered (653 and 570 from 48 and 46 individuals, respectively).

**Table 1:** Summary of the richness of ZOTUs obtained from samples and the specificity of ZOTUs to a particular damselfly species. ‘Unique’ ZOTUs refer to those that were specific to a single damselfly species, regardless of the number of samples in which the ZOTU was detected, while ‘shared’ ZOTUs were observed in samples from more than one species. ZOTU counts are out of a total of 1513. Extrapolated ZOTU total richness estimates are provided with standard errors. The average numbers of damselfly individuals from which particular ZOTUs were recorded are provided with bias-corrected and accelerated 95% confidence intervals

Species	*n*	ZOTU richness	Proportion unique ZOTUs	Extrapolated estimate of total ZOTU richness (SE)		Average number of samples in which a ZOTU was observed (95% CI)	
				Chao	Jackknife	Unique ZOTUs	Shared ZOTUs
*Coenagrion hastulatum*	48	653	0.29	1264.01 (95.63)	982.98 (65.68)	1.29 (1.21–1.41)	4.50 (4.05–5.04)
*Coenagrion lunulatum*	46	480	0.16	957.67 (87.71)	724.57 (67.8)	1.09 (1.03–1.21)	3.67 (3.26–4.08)
*Coenagrion pulchellum*	46	570	0.33	1772.09(200.84)	945.65(124.43)	1.03 (1.01–1.07)	3.00 (2.71–3.43)
*Enallagma cyathigerum*	45	914	0.51	2083.83 (141.42)	1487.96 (237.75)	1.18 (1.14–1.23)	3.48 (3.48–3.98)

### Characterization of ZOTUs Across Damselfly Species: Overall, and of Those Most Frequently Sampled ZOTUs

All four host species showed strong representation by *Gammaproteobacteria* (species average 69–79%) and *Bacilli* (species average 18–25%), moderate representation of *Alphaproteobacteria* (species average 3–7%), *Betaproteobacteria* (species average 2–4%), and *Actinobacteria* (species average 2–5%), and weaker, more sporadic representation of *Mollicutes* (species average 21% at *E. cyanthigerum*), *Negativicutes* (species average 15% at *C. pulchellum*), *Sphingobacteria* (species average 3% at *C. pulchellum*), and *Cytophagia* (species average 1–2%).

**Fig. 2:**
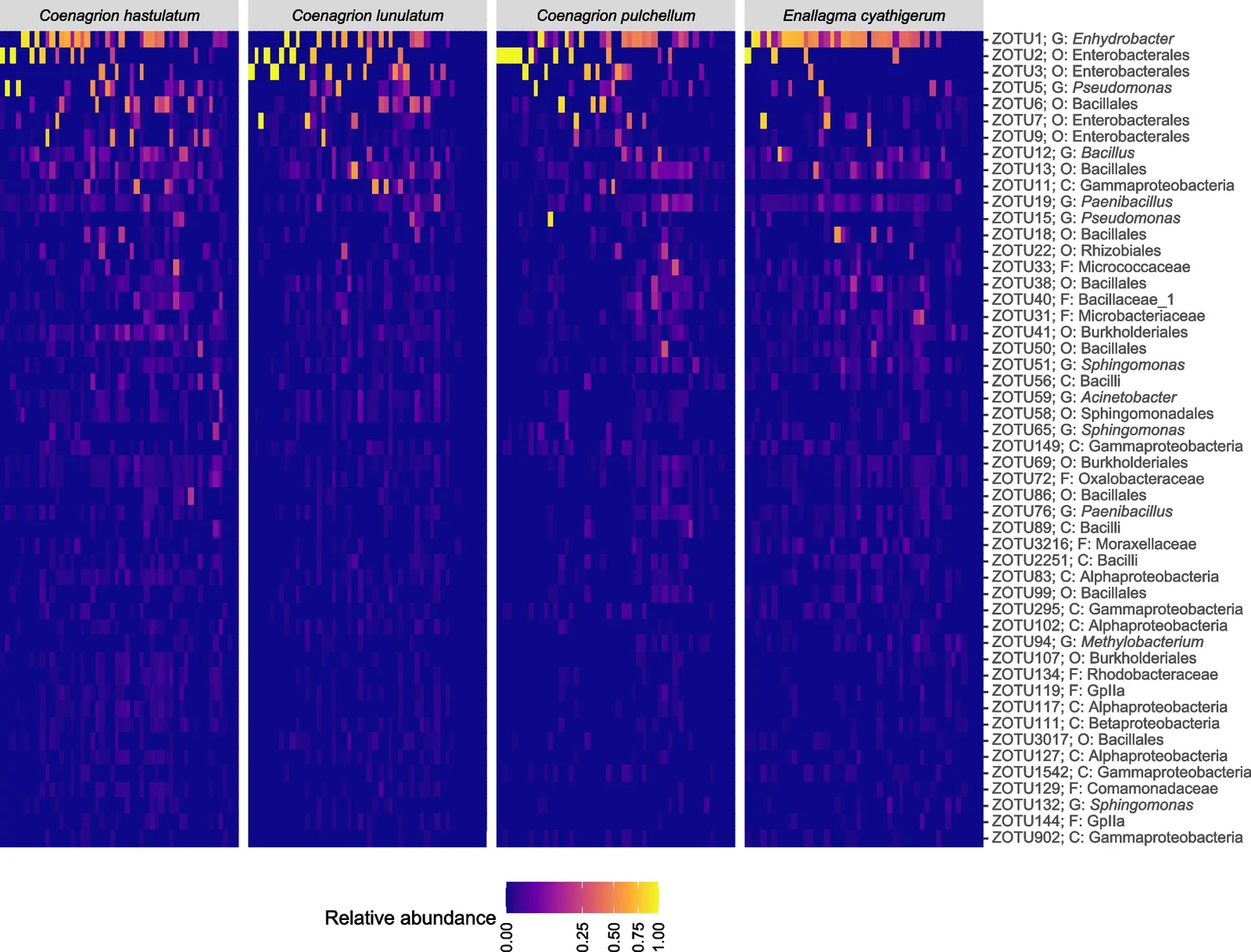
Heatmap of relative read abundances (RRAs) for the 50 most core ZOTUs (those present in the highest proportion of samples overall). Each column represents a single sampled individual (ordered horizontally by variance in RRAs), while each row is a distinct ZOTU (ordered vertically by total RRA). ZOTU IDs are provided on the right, along with the most specific taxonomic level to which they were assigned with a minimum probability of 70% (though these were kept no more specific than genus, even if species was attributed a probability greater than 70%: D, domain; P, phylum; C, class, etc.). The colour scale is mapped to RRA values given a square root transformation to help emphasize the lower RRA values, as values were positively skewed overall

The assigned identities of those 50 ‘most core’ ZOTUs (those detected in the highest proportion of samples across all damselfly species combined) are presented in Fig. 2, along with a heatmap demonstrating their relative read abundances (RRAs) across samples. We found that those ZOTUs with higher RRA values appear to be somewhat more present across samples and present in more damselfly species. There is also initial evidence from Fig. 2 that several ZOTUs do appear to be more prevalent in some species than others, leading to some expected degree of differentiation.

### More Single-Damselfly Species ZOTUs, and Fewer Four-Species ZOTUs, Than Expected by Chance

The number and proportion of species-specific ZOTUs differed among the four damselfly species. For example, 51% (or 466) of 914 ZOTUs recovered from *E. cyathigerum* were unique to that species, compared to 33% (or 188) of 570 ZOTUs for *C. hastulatum*, to 29% (or 189) of 653 ZOTUs for *C. pulchellum*, to ~16% (or 78) of 480 ZOTUs for *C. lunulatum.* This left 488 ZOTUs remaining for *E. cyathigerum* shared with one or more other species, compared to 465 shared ZOTUs remaining for *C. hastulatum*, compared to 381 shared ZOTUs remaining for *C. pulchellum*, and compared to 402 shared ZOTUs remaining for *C. lunulatum*. Intriguingly, the average number of individuals in which a unique ZOTU was found approximated one individual for each unique ZOTU of each of the four host species (Table 1). That is, ZOTUs unique to host species were very incidentally sampled in those host species and not sampled from other species.

**Fig. 3:**
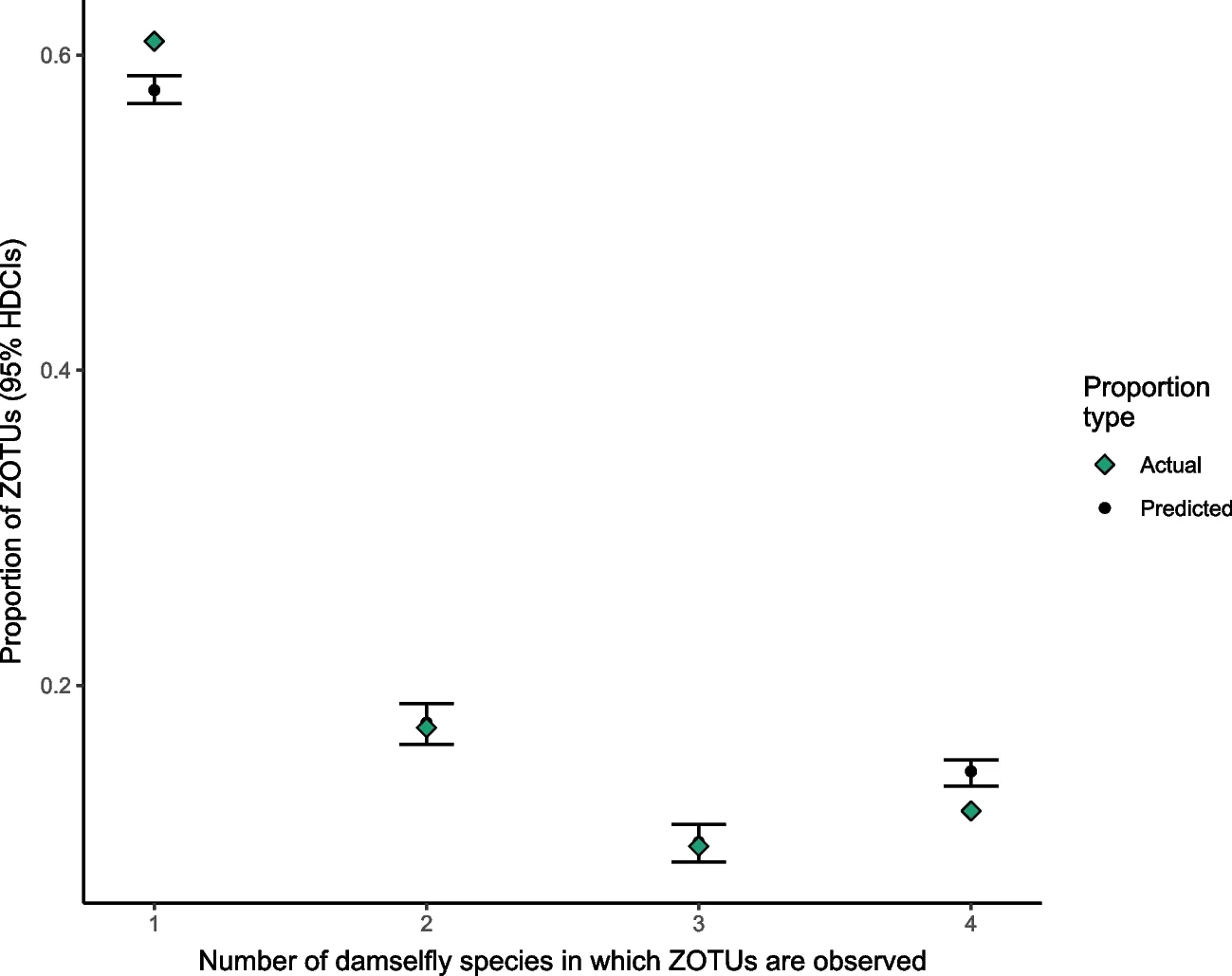
Proportions of the 1513 ZOTUs expected to be specific to one or more of the damselfly species based on 1000 randomizations of the occurrence matrix, compared to observed proportions. The randomization algorithm maintained the number of ZOTU occurrences within each sampled individual, as well as the number of samples in which each ZOTU was observed (maintained both column and row sums of the occurrence matrix). Error bars represent 95% highest density continuous intervals (HDCIs) calculated from the randomizations

**Fig. 4:**
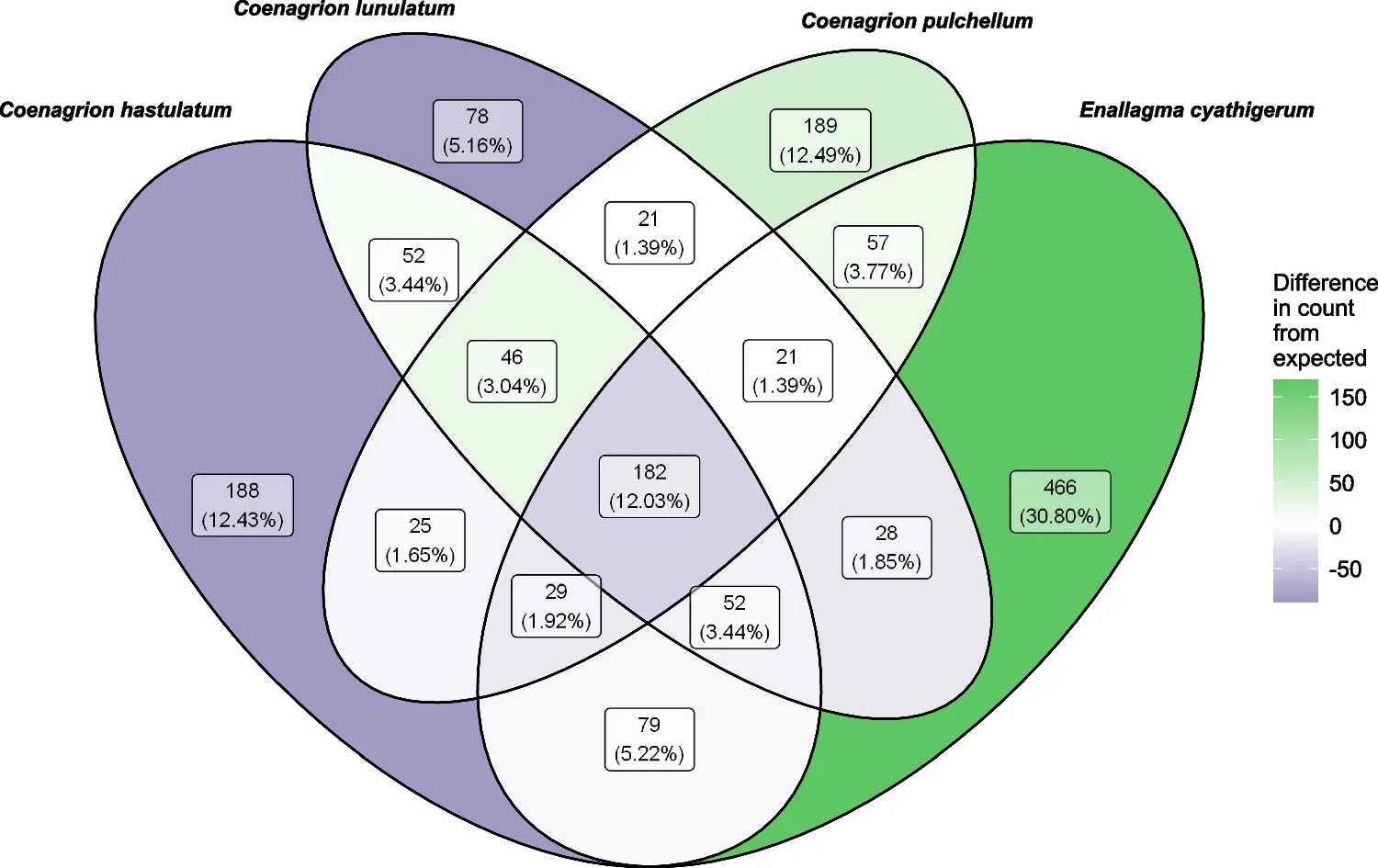
The number and percentages of the 1513 ZOTUs that were observed in samples from each species of damselfly, as well as from each combination of species. The colour of each Venn diagram cell represents the difference between the number of ZOTUs observed and the average that was expected based on 1000 randomizations of the ZOTU occurrence matrix (green indicates a higher count than expected, blue indicates a lower). The randomizations maintained the number of ZOTU occurrences within each sampled individual, as well as the number of samples in which each ZOTU was observed (maintained both column and row sums of the occurrence matrix). The different areas of the cells do not represent anything relating to the data

A total of 921 (or 60.8%) of 1513 distinct ZOTUs were unique to any given species. Grossly, this means that *ca.* 39% of distinct ZOTUs were shared by two or more species. Comparisons of observed single- and multi-damselfly species ZOTUs to null models revealed that the number of species in which ZOTUs occurred did not often match what was predicted by chance when respecting null model constraints (matrix randomizations maintaining both the number of ZOTU presences within samples and the frequency of occurrences of a particular ZOTU across samples). Single-damselfly ZOTUs were more frequent than predicted, while ZOTUs present in all four species were significantly less frequent than predicted by chance (Fig. 3; 95% CIs exclude observed values). When looking at the richness of ZOTUs within specific damselfly species or combinations of species, a more nuanced pattern emerged: there were many more *E. cyathigerum*-specific ZOTUs than expected by chance, but somewhat fewer *C. hastulatum*- and *C. lunulatum*-specific ZOTUs than expected (and slightly more *C. pulchellum*-specific ZOTUs than expected; Fig. 4). This huge surfeit of *E. cyathigerum*-specific ZOTUs likely accounts for the higher-than-expected number of single-species ZOTUs, generally speaking (despite deficits in two species; Fig. 4).

### Differences in ZOTU Assemblage Composition and in Individual ZOTU Representations Between Species

The test for multivariate homogeneity of dispersions in Bray–Curtis dissimilarities among the four species failed (the test showed a significant difference; *F*_3,173_ = 3.77, *p* = 0.012). In other words, while the PERMANOVA comparing ZOTU community composition between the species was significant (though it explained little variance between the species; pseudo-*F* = 2.53, *p* = 1.00 × 10^−4^, *R*^2^ = 0.042), this could not be confidently attributed to differences between the group centroids. The species differ in their within-group interindividual dissimilarities, but the ZOTUs within the species’ assemblages may not greatly differ.

**Fig. 5:**
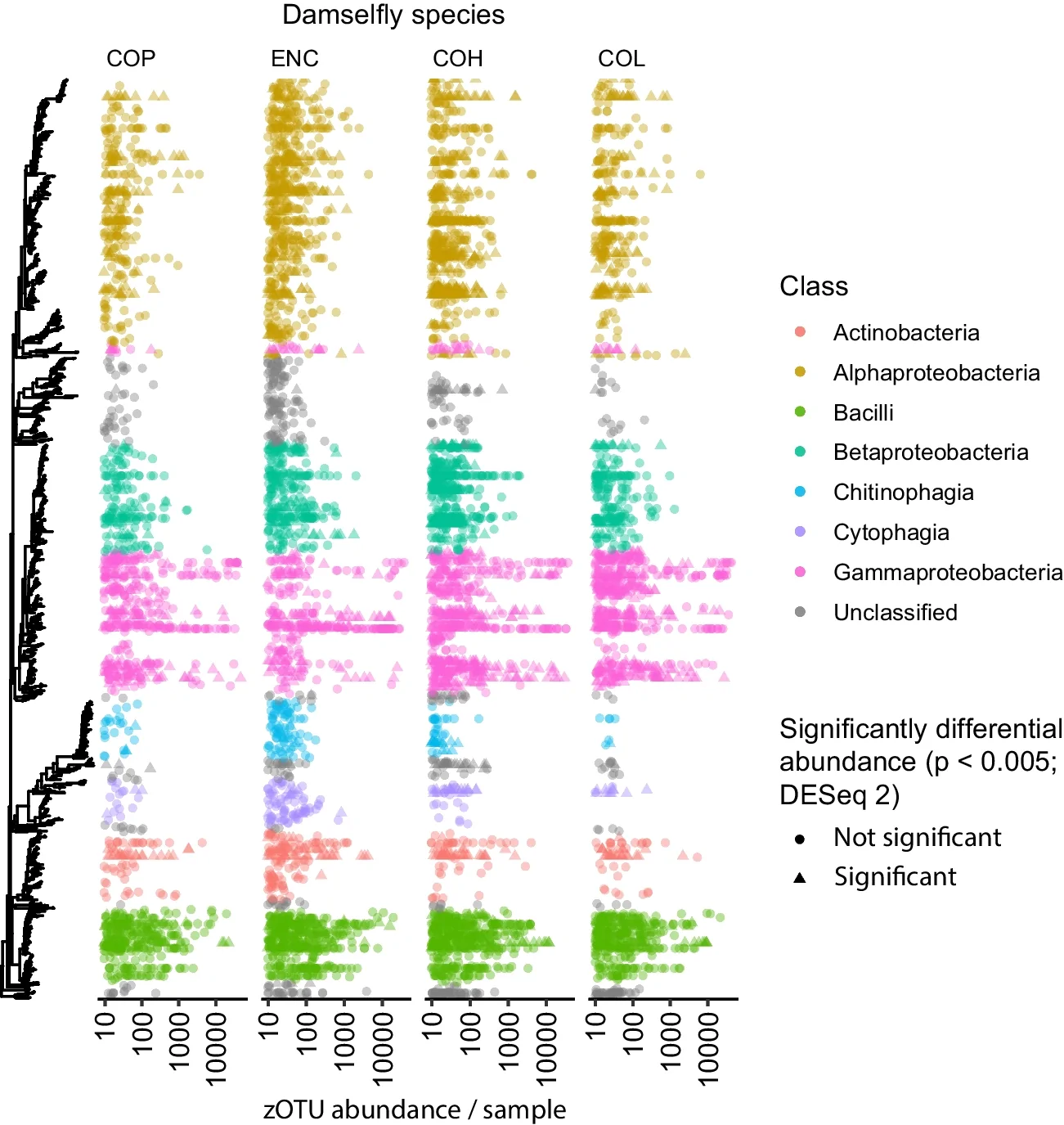
Phylogeny of the damselfly faecal bacteria included in this study (left most panel) and the abundance distribution of the bacterial classes across the four host species. Bacterial ZOTUs coded with a circle did not show significant variation in relative read abundance across the four damselfly species according to DESeq2 analyses (*p* < 0.005), whereas bacterial ZOTUs coded by a triangle showed significant variation across the four host species. Each point represents the absolute read abundance for a bacterial ZOTU in an individual sample of a given host species, presented on a logarithmic scale (columns, COP, *Coenagrion pulchellum*; ENC, *Enallagma cyathigerum*; COH, *Coenagrion hastulatum*; and COL, *Coenagrion lunulatum*)

**Fig. 6:**
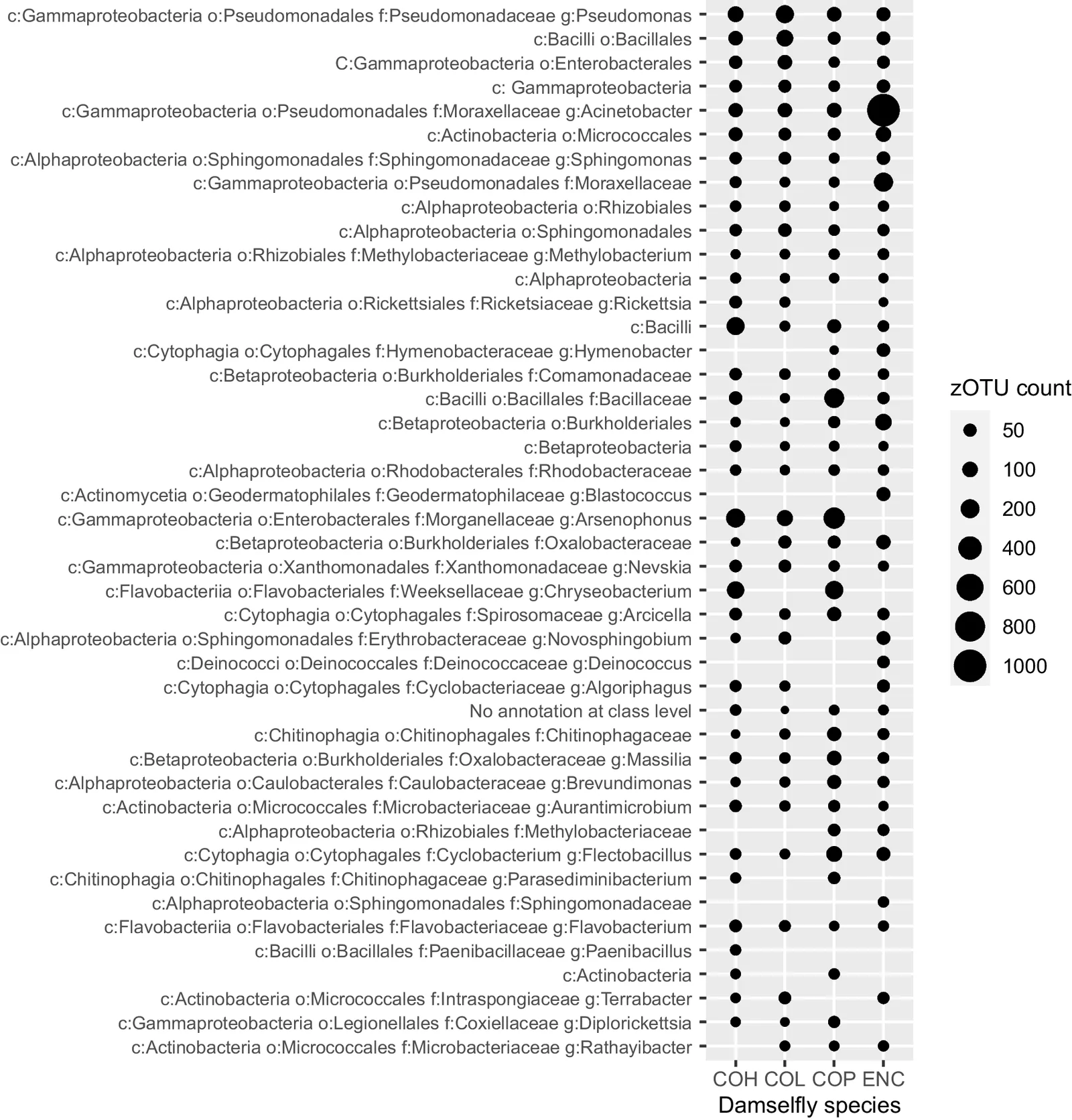
Phylogenetic classification of the significantly variable ZOTUs between the damselfly species (*p* < 0.005; DESeq2). Classification is provided for the class, order, family and genus levels whenever available (indicated as c, o, f and g in the labels, respectively). Relative sizes of the visualized circles represent the number of reads for each significantly variable ZOTU in each damselfly species (columns, COP, *Coenagrion pulchellum*; ENC, *Enallagma cyathigerum*; COH, *Coenagrion hastulatum*; and COL, *Coenagrion lunulatum*)

DESeq2 analyses identified a set of 145 ZOTUs from the total of 1513 ZOTUs with apparent differential abundance between the four damselfly species (DESeq2; *p* < 0.005), which were dispersed throughout the phylogeny and consisted of 44 distinct taxonomic profiles (Fig. 5). Of these differentially expressed ZOTUs, some of the most separated ones occurred in *E. cyathigerum*, including an overrepresented *Acinetobacter* genus, exclusive *Deinococcus* and *Blastococcus* genera, and *Sphingomonadaceae* families (Fig. 6). Recalling the overrepresentation of non-shared ZOTUs observed in *E. cyathigerum* in the earlier analyses, it is notable that, of those ZOTUs indicated as significantly differentially abundant by DESeq2, the only three that were specific to a single species were observed in *E. cyathigerum* (Fig. 6). Other seemingly differentiated ZOTUs included an *Actinobacterial* class and *Parasediminibacterium* and *Chryseobacterium* genera exclusively present in *C. hastulatum* and *C. pulchellum*, and *Methylobacteriaceae* family and *Hymenobacter* genera exclusively present in *C. pulchellum* and *E. cyanthigerum* (Fig. 6).

## Discussion

The comparison of bacterial assemblages across host species, combined with explicit treatment of bacterial phylogeny, can provide valuable insights into the ecological and evolutionary forces that govern the diversity and differentiation of these associations [46–49]. In this study, our general approach was to compare distinct bacterial species that are shared versus not shared between host species with respect to sampling likelihood, null expectations of (co-)occurrence versus actual (co-)occurrence in different host species, and taxonomic or phylogenetic representation of the bacteria themselves. We found that one damselfly species’ faecal bacterial assemblage appears to be particularly rich with an overabundance of non-shared ZOTUs, and that while there is some evidence for differential representation of bacterial taxa among the damselflies, significantly differing representations of ZOTUs were not prevalent among the many taxa which were detected.

Our study highlights that damselfly faecal bacterial assemblages are diverse, with 1513 distinct ZOTUs across all four focal species, which is likely an underestimate based on species accumulation curves. In a previous study of gut-associated bacteria of Odonates, Deb et al. [21] reported a much lower number (567) of ZOTUs. In this previous study, the bacteria were isolated from dissected gut tissue, whereas in our study, the bacteria were isolated from faeces. We suspect that the disparity in total richness when comparing our results with those of Deb et al. 2019 was the result of our having odonate sample sizes that were between three and five times as large as theirs, especially given that ZOTU accumulation curves were still increasing. Furthermore, the use of insect gut tissue rather than faecal samples can influence the yield and diversity of extracted microbial DNA as well [50], and further research is required to understand the contribution of sample types to the observed composition of odonate bacterial assemblages. Notwithstanding, we predicted little difference in assemblages between damselfly species as these are generalist predators (e.g. [28]) occupying the same location with identical availability of prey, at the same timing of sampling. However, we found some differences in richness of faecal bacterial assemblages among damselfly species, seemingly accounted for by among-host variation in occurrence of non-shared bacteria.

When we compared shared vs. non-shared ZOTUs, one of the four focal species—*Enallagma cyathigerum*—stood out from the rest. We do not know exactly why *E. cyathigerum* had so many rarely sampled non-shared ZOTUs, but this also accounted for the finding of overrepresentation of species-specific ZOTUs in our dataset. In our previous paper [28], we compared the dietary composition of these same four damselflies, and according to these results, *Enallagma cyathigerum* did not have more variable diet than the rest of the focal odonate species, which indicates that diet does not straightforwardly explain the higher number of non-shared ZOTUs of *E. cyathigerum*. Perhaps the longer flight period and/or larger population size of *E. cyathigerum*, as compared to the other three focal species, affected the results [28]. These hypothetical explanations are not mutually exclusive and would require empirical verification. The species-specific ZOTUs are unlikely to represent anything of functional significance, but they do contribute to overall richness estimates. They are so infrequently encountered that they likely often just account for ‘noise’ in terms of characterization of bacterial assemblages. Still, the faecal bacterial assemblages were not simply neutral reconstructions from a single general assemblage, in part because of the overrepresentation of single species ZOTUs, but also because there were many fewer ZOTUs shared between all four species than expected based on a null model, even correcting for a sparse matrix of host species-by-ZOTUs. This added deviation from neutral assembly implies that bacterial assemblages are at least somewhat differentiated among host species with respect to frequently sampled ZOTUs. Partial differentiation of bacterial assemblages is further seen in the heat map results of core bacteria shared between species, but seemingly unevenly so in many cases. Also, the DEseq2 analysis suggests partial differentiation when considering individual ZOTUs, but in most of those instances, the ZOTUs of interest still occurred in the four damselfly species (Fig. 6), and variation in raw reads among samples may not relate to biological differences of interest. These results combined with the fact that *ca*. 39% of frequently sampled bacterial ZOTUs were shared make a case for limited differentiation in bacterial assemblages, among these species of damselflies. Testing whether shared bacteria have functional significance to their damselfly hosts was beyond the scope of this study, though would be a valuable next step. Additionally, determination of which ZOTUs are truly shared across, or specific to, damselfly species may require more in-depth studies involving molecular characterization not based on ZOTUs to confirm whether bacteria deemed shared are truly shared or cryptically distinct.

In studies such as this one, it is important to consider how bacteria can be included in samples. They could, for instance, be obligate or facultative symbionts, maternally transferred, or they could be ingested with prey, imbibed with water, or even breathed in [7, 16, 46, 48, 51]. We decided to include cyanobacteria found in our samples in our analyses. In fact, these occurrences are no different than other bacteria ingested incidentally (e.g. with prey). It is a challenge to determine the extent to which different modes of acquisition have different signals in the data (e.g. high or low prevalence, high or low read abundance, widespread sharing versus host specificity). The possibility of co-ingestion with prey operating as a primary mode of bacterial acquisition could be assessed with faecal metabarcoding approaches as well, by testing for consistent associations in individual samples between one or more bacterial ZOTUs and particular prey taxa. Differential representation of particular bacterial taxa may then be identified as arising due to differences in feeding preferences among the syntopic species and differences in feeding which may be more difficult to identify without molecular approaches to the classification of prey. On the other hand, bacteria which have no presumed single route of entry into the damselfly and, in particular, those which additionally have no functional significance should have less predictable occurrences. For those researchers interested in the possibility of conserved obligate gut symbionts, a good place to start might be with frequently sampled, represented over the season, shared ZOTUs of classes which have been known to include genera that are insect gut symbionts.

Often, insect gut bacterial assemblages are characterized based on the phyla or classes represented. For example, in a study of flesh fly larvae and adults of various species in the family Sarcophagidae, bacteria from the phyla Proteobacteria, Actinobacteria, Firmicutes, and Bacteroidetes were commonly found using barcoding of 16S rRNA gene sequences from gut tissue [52]. In another study of Black soldier flies, the gut bacteria most well represented were found in the classes Gammaproteobacteria and Bacilli (Phyla Proteobacteria and Firmicutes, respectively) and selected representatives were thought to play important roles in this insect’s nutrition [53]. In one study of a cockroach, phyla of bacteria well represented in the gut included Bacteroidetes, Firmicutes, Proteobacteria, and Synergistetes with an increased presence of Firmicutes in guts of sugar-cane-fed insects because of the requirement for lignocellulose digestion [54]. Such information collectively provides a needed reference for comparative studies on bacterial assemblages between insect taxa and provides a clue as to how flexible the insect gut bacterial assemblage is within species.

Previous genetic work to date on gut-associated bacteria of odonates [e.g. 22, 23] was work on dragonflies which showed strong representation of the classes Alphaproteobacteria and Gammaproteobacteria, moderate representation of the class Bacilli, and weaker representation of Actinobacteria, which parallels our data. It is also striking that the proportional representation of bacterial classes was similar between the host species included in this study, again suggesting little differentiation of bacterial assemblages at this phylogenetically informed higher taxonomic level. It is tempting to think of shared bacteria as more likely representing gut-associated bacteria than non-shared bacteria, and in our study, the classes Gammaproteobacteria, Bacilli, and Alphaproteobacteria were seen as sources of shared ZOTUs. Increasing our knowledge of, and confidence in, the taxonomic representation of core, shared bacteria in damselflies as well as odonates more broadly will have potential conservation significance, as healthy populations like those studied herein (stable, sampled as mature adults, and having high abundances; [28]) may provide comparison points for other populations experiencing disease outbreaks or alternative causes of decline.

In conclusion, there are little differences in richness and little differentiation of faecal bacterial assemblages among syntopic damselfly species. Small differences in richness that exist appear mostly due to a surfeit of non-shared bacteria in one damselfly species. Limited differentiation of shared bacteria occurs among species for unknown reasons, but this is overshadowed by the sheer magnitude of ZOTU sharing between species and the little differentiation that exists at a phylogenetically informed higher taxonomic level. The extent to which faecal bacteria represent gut-associated bacteria remains an open question, but our data suggest that bacterial ZOTUs shared between host species might be a useful first approximation. Future work should focus on spatiotemporal and life history stage variation in ZOTU representation and identify those bacterial species persistent through space and time, as possible candidates for obligate symbionts. Whereas high prevalence and sharedness of ZOTUs between species could indicate candidate bacteria of functional significance, the potential functional significance of non-shared bacteria may also be worth investigating in cases where these contribute to higher microbial richness, at least where these ZOTUs are also prevalent in the single host.

## Data Availability

Labelled raw FASTQ reads, ZOTUs, ZOTU assignations, and ZOTU tables are publicly available in the Dryad Digital Repository: 10.5061/dryad.08kprr58q.
